# Detection and Genomic Characterization of Torque Teno Virus in Pneumoconiosis Patients in China

**DOI:** 10.3390/v16071059

**Published:** 2024-06-30

**Authors:** Xiao-Wei Yu, Qiong Wang, Lang Liu, Zhi-Jian Zhou, Tuo Cai, Hua-Ming Yuan, Mei-An Tang, Jian Peng, Sheng-Bao Ye, Xiu-Hong Yang, Xiao-Bin Deng, Xing-Yi Ge

**Affiliations:** 1 Hunan Prevention and Treatment Institute for Occupational Diseases, Changsha 410003, China; yuxw0714@163.com (X.-W.Y.); liul3219@163.com (L.L.); dream_catu2023@163.com (T.C.); yuccnhnszfy@sina.com (H.-M.Y.); tmeianc920@163.com (M.-A.T.); pengj8620@163.com (J.P.); yxh502@sina.com (X.-H.Y.); 2 College of Biology, Hunan Provincial Key Laboratory of Medical Virology, Hunan University, Changsha 410082, China; zjzhou@hnu.edu.cn (Z.-J.Z.); yeshengbao@hnu.edu.cn (S.-B.Y.); 3 Department of Basic Biology, Changsha Medical University, Changsha 410219, China; qw@hnu.edu.cn; 4 Hunan Provincial Key Laboratory of the Traditional Chinese Medicine Agricultural Biogenomics, Changsha 410219, China

**Keywords:** pneumoconiosis, TTV, genome, phylogenetic analysis

## Abstract

Pneumoconiosis is a common occupational disease that can worsen with accompanying infection. Torque teno virus (TTV) is a prevalent human virus with multiple genotypes that can chronically and persistently infect individuals. However, the prevalence of TTV in pneumoconiosis patients is still unclear. This research aims to detect the presence and prevalence of TTV in the alveolar lavage fluid of pneumoconiosis patients in the Hunan Province of China using PCR. As a result, a 65.5% positive rate (19 out of 29) of TTV was detected. The TTV detection rate varies among different stages of silicosis and different pneumoconiosis patient ages. Nine novel TTV genomes ranging in size from 3719 to 3908 nt, named TTV HNPP1, HNPP2, HNPP3, HNPP4, HNPP5, HNPP6-1, HNPP6-2, HNPP7-1 and HNPP7-2, were identified. A genomic comparison and phylogenetic analysis indicated that these nine TTVs represent five different species with high genetic diversity which belong to the genus *Alphatorquevirus*. HNPP6-1 and HNPP6-2 belong to TTV3, HNPP5 belongs to TTV13, HNPP1 belongs to TTV24, HNPP4 belongs to TTV20, and the others belong to TTV19. The genomes of TTV HNPP1, HNPP6-1, and HNPP6-2 contain three putative open reading frames (ORFs) coding for proteins, ORF1, ORF2, and ORF3, while the other six TTV genomes contain two ORFs coding for proteins, ORF1 and ORF2. These results provide the first description of TTV epidemiology in pneumoconiosis patients in China. The newly identified TTV genome sequences reveal the high genetic diversity of TTV in pneumoconiosis patients and could contribute to a deeper understanding of TTV retention and infection in humans.

## 1. Introduction

Torque teno virus (TTV) is a small, non-enveloped virus with a single-stranded circular DNA genome that belongs to the genus *Alphatorquevirus* within the family *Anelloviridae* [1]. The TTV genome is around 3.8 kb in size and contains a GC-rich untranslated region (UTR) and two major open reading frames (ORFs), named ORF1 and ORF2, with putative additional ORFs among different isolates. Overlap between the ORFs is common, and their estimated sizes differ widely among isolates [2]. The putative ORF1 is the longest ORF of TTV, covering approximately two-thirds of the entire viral genome. Currently, based on the molecular phylogeny of ORF1, the *Alphatorquevirus* genus consists of 26 species, including 22 species infecting humans and 4 species infecting non-human primates [3,4,5]. TTV is transmitted through various routes of infection, including fecal–oral, parenteral, sexual and vertical transmission, resulting in its widespread occurrence in different populations and regions [6,7]. However, the global prevalence of TTV genotypes is highly diverse, and the clinical consequences of TTV infection remain unclear.

In 1997, TTV was first identified in a case of post-transfusion hepatitis with an unknown etiology in a human, leading to suspicions of its connection to liver disease [8]. Subsequently, numerous studies have proposed its association with various hepatic disorders such as acute, chronic, and fulminant hepatitis. Furthermore, TTV has been detected in patients with conditions such as thalassemia, hemodialysis, viremia, pulmonary fibrosis, and idiopathic inflammatory myopathies. The prevalence of TTV is significantly higher in patients with severe disease compared to those with mild disease [9,10,11]. On the other hand, co-infections of TTV with other viruses have been extensively investigated. Studies have reported that hepatitis patients co-infected with TTV have a higher mortality rate and may be more likely to develop hepatocellular carcinoma compared to patients infected with hepatitis B virus or hepatitis C virus alone [12,13,14]. Additionally, TTV was detected in patients’ saliva during the SARS-CoV-2 epidemic [15,16,17]. Recent studies suggested anellovirus as an ideal candidate for next-generation gene drug delivery based on its characteristics [18]. Although there is no pathological evidence directly linking TTV to the onset and progression of the disease, its presence in various diseases and its genome characterization merit investigation.

Pneumoconiosis is a condition caused by inhaling mineral dust into the lungs, resulting in lung fibrosis and silicosis. Pneumoconiosis has recently become a significant public health issue due to its global prevalence and higher mortality rate [19,20]. Additionally, pneumoconiosis patients often experience co-infections with bacteria and viruses which worsen the disease and make treatment more difficult. According to previous studies, the positive rates of TTV in diseased and healthy populations are 2–90% [21,22]. However, there have been no studies on TTV in patients with pneumoconiosis. In this study, we collected alveolar lavage fluid samples from pneumoconiosis patients, revealing the presence of TTV in these samples. We also characterized and analyzed the genomes of nine novel TTV variants. This is the first report of TTV detection in pneumoconiosis patients with a complete genome analysis, and our findings indicate that TTV is widespread in pneumoconiosis patients and exhibits a high degree of diversity. Therefore, more attention should be paid to TTV in pneumoconiosis patients to explore its correlation with diseases.

## 2. Materials and Methods

### 2.1. Ethics Statement

Sample collection and all experiments in this study received ethics approval from the Hunan Prevention and Treatment Institute for Occupational Diseases, with the ethics approval number LLYS2019008. All patients participating in the tests were verbally informed of the purpose of collecting the samples and gave permission for their use.

### 2.2. Sample Collection

From 2019 to 2021, 29 alveolar lavage fluid samples were obtained from male pneumoconiosis patients in the Hunan Province of China, including 16 patients with silicosis stage 1, 7 patients with silicosis stage 2, and 6 patients with silicosis stage 3, during routine surveillance of pathogen control. All samples were stored on dry ice for short-term storage and then transported to the laboratory and stored at −80 °C until use.

### 2.3. DNA Extraction and PCR Screening

Viral DNA was extracted from 200 μL alveolar lavage fluid samples using a TIANamp Virus DNA/RNA Kit (Tiangen, Beijing, China), following the manufacturer’s instructions, and stored at −80 °C. To screen for the presence of TTV DNA, a PCR was performed using primers derived from the 5′UTR-based primers as described previously (NG133: 5′-GTAAGTGCACTTCCGAATGGCTGAG-3′; NG147: 5′-GCCAGTCCCGAGCCCGAATTGCC-3′) [23]. A PCR amplification was performed using the Ex Taq^®^ DNA Polymerase (Takara, Beijing, China). The PCR mixture (25 μL) contained 2 μL of extracted DNA, 2.5 μL of 10 × Ex Taq Buffer (20 mM), 2.0 μL of dNTP Mixture (2.5 mM each), 0.4 μM of NG133/NG147 primers and 0.125 μL of Ex Taq DNA polymerase (5 U/μL). The mixtures were amplified by 35 cycles of denaturation at 94 °C for 30 s, annealing at 60 °C for 30 s and extension at 72 °C for 30 s.

The PCR products were gel-purified and cloned into a T-Vector, and at least 3 positive clones of each fragment were sequenced using the Sanger method. The sequences of the PCR products were compared with known sequences of TTV deposited in the GenBank database.

### 2.4. Complete Genome Sequencing

After PCR screening and sequencing, samples which were singly positive for TTV were selected to amplify the complete genomes. A nested long-distance PCR was performed using primers derived from the 5′UTR- and 3′UTR-based primers (NG133/NG135 for the first round and NG134/NG136 for the second round) as described previously [23]. The PCR amplification was performed using the PrimeSTAR^®^ GXL DNA Polymerase (Takara, Beijing, China). The first round of amplification was carried out in a total reaction volume of 25 μL using 1 μL of extracted DNA, 5 μL of 5 × PrimeSTAR GXL Buffer, 2.0 μL of dNTP Mixture (2.5 mM each), 0.4 μM of NG133/NG135 primers and 0.5 μL of PrimeSTAR GXL DNA Polymerase for 35 cycles. Each cycle consisted of denaturation at 98 °C for 10 s, annealing at 60 °C for 15 s, extension at 68 °C for 3 min 30 s and a final extension at 68 °C for 10 min. The second round was performed with 0.4 μM of NG134/NG136 or NG149/NG132 using 1 μL of the round 1 PCR product diluted 100 times as a template for 35 cycles of the same cycling parameters as in the first round. The PCR products were gel-purified and cloned into the T-Vector, and at least three positive clones of each fragment were sequenced by Sanger method. The obtained sequences were used to assemble the viral genomes.

### 2.5. Genomic and Phylogenetic Analysis

The ORF Finder (National Center for Biotechnology Information) was used to predict potential open reading frames (ORFs) and their corresponding amino acid (aa) sequences. The gene sequences and the encoded protein aa sequences were compared to those of known viruses with complete genomes available in GenBank. MEGA7 was used to generate and edit sequence alignment [24]. The alignment datasets were used to generate the phylogenetic trees by IQ-TREE 2 in BioAider, using the maximum likelihood (ML) method with 1000 ultrafast bootstraps, and the most appropriate substitution aa model was determined using ModelFinder according to the Bayesian information criterion (BIC) method [25].

## 3. Results

### 3.1. TTV Detection in Pneumoconiosis Patients

To confirm the prevalence of TTV in pneumoconiosis patients, we performed a PCR screening of 29 alveolar lavage fluid samples using primers designed in the 5′UTR conserved region. The results showed that 19 out of the 29 samples tested positive for TTV, resulting in an overall positivity rate of 65.5% (Table 1). Further grouping of pneumoconiosis patients based on age and silicosis stage revealed that the TTV detection rates of pneumoconiosis patients with silicosis stages I, II and III were 52.3%, 71.4% and 83.3%, respectively, and the TTV detection rates of pneumoconiosis patients aged ≤50, 51–60 and >60 were 62.5%, 64.7% and 75%, respectively (Table 1). These results indicate that TTV is widespread among pneumoconiosis patients, and the TTV detection rate varies among different stages of silicosis and different pneumoconiosis patient ages.

### 3.2. Genome Characterization of Novel TTVs

**Figure 1:**
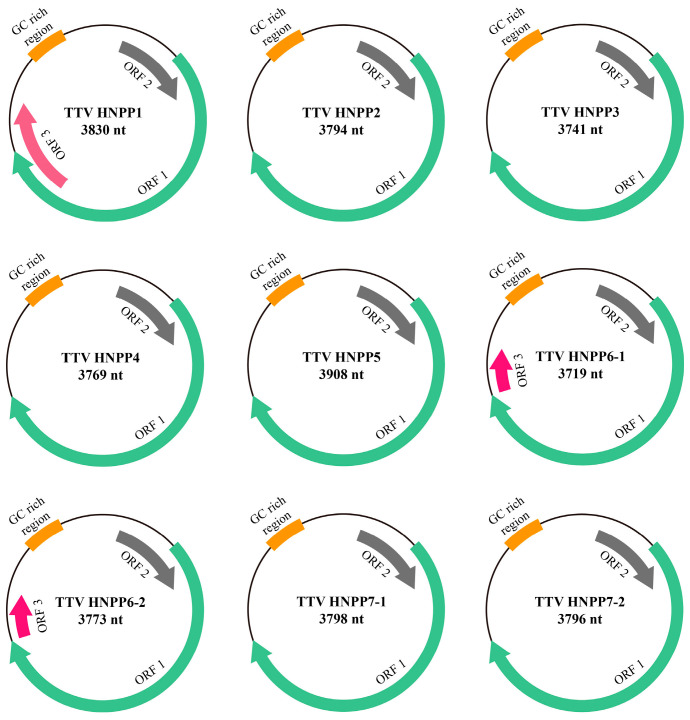
Genome organizations of TTVs characterized in this study. Arrows indicate predicted coding sequences and transcription directions. The orange sections refer to the GC rich region.

To elucidate the genomic characterization of TTV in pneumoconiosis patients, six samples showing strong positivity for TTV were chosen to amplify the complete genomes. The replicons were cloned, sequenced and assembled to obtain the complete genomes. Notably, three out of the six positive samples were able to amplify two different TTV strains each, resulting in nine complete TTV genomes. The genomes of TTV HNPP1, TTV HNPP2, TTV HNPP3, TTV HNPP4, TTV HNPP5, TTV HNPP6, TTV HNPP6-1, TTV HNPP7-1, and TTV HNPP7-2 obtained in this research are 3830 nt, 3794 nt, 3741 nt, 3769 nt, 3908 nt, 3719 nt, 3773 nt, 3798 nt, and 3796 nt in size, respectively (Figure 1). All genomic sequences have been deposited in GenBank with the accession numbers: PP374828-PP374836.

### 3.3. Analysis of Genomes of Novel TTVs

The circular genomic DNA of the nine novel TTV strains displays a range of lengths from 3719 nt to 3908 nt, with a GC content ranging from 50.7% to 53.4%, respectively (Table S1). The genomes were annotated via BLASTx and BLASTp searches, and two typical open reading frames (ORF1 and ORF2) of the nine novel TTVs were identified. The two ORFs were aligned as 5′UTR-ORF1-ORF2-3′UTR. Moreover, an additional ORF3 was identified in HNPP1, HNPP6-1, and HNPP6-2. As shown in Figure 1, the TTV coding sequences are arranged in a clockwise direction on the same strand of circular single-stranded DNA, which is similar to other TTVs (Figure 1). The accurate locations and lengths of the predicted ORFs, as well as the molecular weights of the corresponding proteins, are listed in Table 2.

The nine novel TTV genomes share several common characteristics with previously reported TTVs. ORF1 is the longest ORF and encodes a protein of 736 aa to 790 aa with an arginine-rich N-terminus, conserved amino acid motifs, and a downstream poly-adenylation signal. ORF1 is predicted to encode the putative capsid and replication-associated protein [26]. Notably, the N-terminal region of ORF1, which contains amino acids that can form multiple motifs conforming to K/R-X-X-R (X refers to an arbitrary amino acid) is hypothesized to be cleaved by furin, thereby activating the activity of ORF1. Rolling circle replication (RCR) is the mode of replication of most ring DNA viruses. All TTV ORF1s (except TTV HNPP4) were found to have the RCR motif III: Y-X-X-K. The conserved WGGN motifs may be associated with putative helicase activity. Additionally, conserved motifs such as FYR, KWY and FQVL were discovered in ORF1 (Figure S1, Table S1). ORF2 may encode a protein with phosphatase activity or a peptide capable of suppressing NF-κB pathways [27]. ORF2 of the nine TTVs encodes putative proteins between 153 and 163 aa in the same orientation and overlapping with the N-terminal end of ORF1. This region that contains a highly conserved W-X7-H-X3-C-X1-C-X5-H (CAV-like) motif.

### 3.4. Gene Similarities and Phylogenetics of Novel TTV

We further compared the nt and aa identities of the putative ORF1 for species demarcation between these nine TTVs and other related TTVs. As shown in Table S1, the nt sequences of ORF1 and ORF2 for TTV HNPP6-1 are identical to those of TTV HNPP6-2, respectively, indicating that these two TTVs belong to the same species. TTV HNPP7-1, HNPP7-2, HNPP2, and HNPP3 also showed high nt and aa sequence identities, higher than 88% in ORF1, suggesting that these four strains are from the same virus species. On the other hand, the ORF1 nt identity for the remaining three TTVs, HNPP1, HNPP4, and HNPP5, ranged from 47% to 52%, whereas the corresponding aa identity ranged from 36% to 43%.

**Figure 2:**
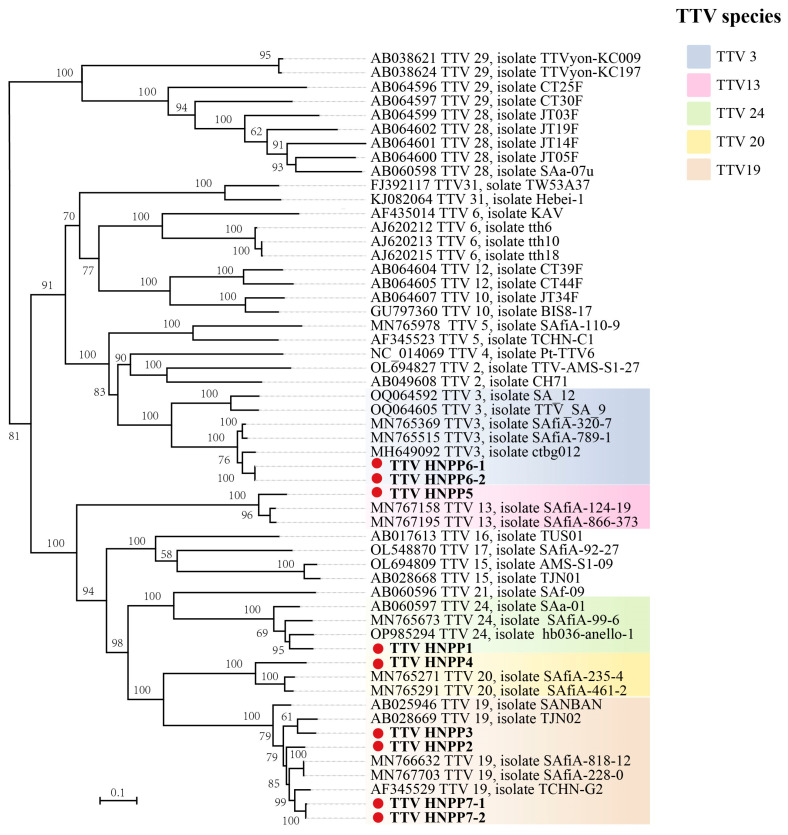
A phylogenetic analysis of human TTV. The tree was constructed based on the alignment of complete nucleotide sequences of ORF1 by the maximum likelihood method with 1000 bootstrap replicates. Bootstrap values above 50% are shown. Yellow indicates TTV 20. The pink square indicates TTV 13. Light brown indicates TTV 19. The cyan square indicates TTV 24. The light blue square indicates TTV 3. For all TTVs included in the tree, GenBank accession numbers and full names are shown. The nine novel TTVs detected in this study are indicated in bold with solid circles.

Based on the nucleotide sequences of ORF1, we constructed a phylogenetic tree using nine TTVs from pneumoconiosis patients and 46 other TTVs from GenBank. The results show that these TTVs were classified into five distinct TTV species. The major branches of the phylogenetic tree were well supported (Figure 2). HNPP6-1 and HNPP6-2 clustered with TTV 3. HNPP1, HNPP4, and HNPP5 were clustered with TTV 24, TTV 20, and TTV 13, respectively. In addition, HNPP2, HNPP3, HNPP7-1, and HNPP7-2 all belonged to the same evolutionary species as TTV 19.

## 4. Discussion

In the *Anelloviridae* family, anelloviruses in the three genera *Alpha*-, *Beta*- and *Gammatorquevirus* have been frequently detected in human samples. The ICTV classified these viruses based on genome size, and they are usually known as Torque teno virus (TTV, 3.9 kb), Torque teno mini virus (TTMV, 2.8–2.9 kb) and Torque teno midi virus (TTMDV, 3.2 kb), respectively [1]. Anelloviruses display extensive genetic diversity and have been detected in human blood and various tissue types. In addition to its immune escape characteristics, annellovirus is a compelling candidate for a next-generation genetic medicine vector. Recently, a gene delivery vector called “Anellovector” was successfully developed based on a human commensal anellovirus [18].

Since the initial report on TTV, studies have indicated its presence in different diseases such as hepatitis, pneumonia, and trans-fusional diseases [15,28]. However, there have been no reports of TTV in pneumoconiosis patients. In this study, we detected the presence of TTV in pneumoconiosis patients for the first time and identified nine complete TTV genomes. A genetic evolutionary analysis of ORF1 showed that these nine TTVs belonged to five different TTV species along with TTV strains that were recently identified. These findings suggest a significant level of genetic diversity of TTV in pneumoconiosis patients.

Even though TTV is a DNA virus, the nucleotide sequence of its genome shows a high degree of divergence; each of the known 22 human TTV species contains various strains [3,5,29]. In this study, according to the most recent TTV categorization, pneumoconiosis patient #5 was co-infected with TTV HNPP7-1 and HNPP7-2, and the nt sequence identities of ORF1 and ORF2 of these two TTVs were higher than 97%, indicating that they belonged to the same species. Pneumoconiosis patient #10 was co-infected with TTV HNPP1 and HNPP2. The nt sequence identities of ORF1 and ORF2 of these two TTVs were 55% and 58%, respectively, and they belonged to distinct TTV species. Furthermore, pneumoconiosis patient #2 was co-infected with TTV HNPP5 and HNPP6-1. However, the nt identities of ORF1 of these two TTV strains were significantly lower, and they belonged to different species. These findings imply that different TTV strains can infect the same pneumoconiosis patient. Further research is needed to investigate the consequences of different TTV genotypes infecting the same patient.

Despite their high genetic divergence, all nine newly identified TTV genomes display a conserved genetic organization. They have a coding region that contains the major open reading frame, ORF1, as well as an overlapping ORF2 and several other ORFs (Table 2). Additionally, these TTV genomes are roughly the same size. It is worth noting that the ORF3 of TTV HNPP1 is 186 aa, whereas the ORF3 of both HNPP6-1 and HNPP6-2 is 59 aa. This may be a result of nucleotide deletions or mutations during TTV gene evolution.

TTV can be detected in different regions and populations, regardless of age, sex, or socioeconomic status. It can reach a prevalence rate of up to 90% in healthy individuals [21,28]. In our study, the positive rate of TTV in pneumoconiosis patients was 65.5%, which is lower than in the general population. This could be related to the small number of lung lavage fluid samples or immune system variability among individuals or different sample types. Additionally, our results preliminary showed that age and silicosis staging had an effect on TTV infection in pneumoconiosis patients. Given the small number of samples collected in this study, comparing the positive rates of TTV in groups may not reflect its true results. In future research, collecting more samples from patients with different ages or stages of silicosis will help to better study the relationship between TTV and pneumoconiosis patients. TTV is almost prevalent worldwide and is insensitive to current antiviral drugs. Many consider TTV a harmless endosymbiont, but there is no direct biological evidence for this. At present, there is a lack of cell and animal infection models for TTV. Future progress and improvement in these areas will promote research on the functions of TTV.

## 5. Conclusions

In summary, our results revealed that TTV is commonly found in the alveolar lavage fluid of pneumoconiosis patients, with an overall prevalence of 65.5%. Furthermore, we discovered nine complete TTV genomes. Through a phylogenetic analysis of ORF1, we determined that these nine TTV isolates belonged to five viral species. However, additional investigation is needed to determine whether these TTVs are directly associated with disease in pneumoconiosis patients.

## Figures and Tables

**Table 1:** Detection of TTV in bronchoalveolar lavage fluid of pneumoconiosis patients with different classifications and ages.

Characteristic		Positive Rate
Stage	I	9/16 (52.3%)
	II	5/7 (71.4%)
	III	5/6 (83.3%)
Age	≤50	5/8 (62.5%)
	51–60	11/17 (64.7%)
	>60	3/4 (75%)
Total		19/29 (65.5%)

**Table 2:** Predicted open reading frames (ORFs) of TTV and molecular masses (kilo Dalton) and isoelectric point (pI) values of potential encoded proteins.

TTV Virus	ORFs	Strand	Position	Length		MW kDa	pI
				nt	aa		
TTV HNPP1	ORF1	+	615–2915	2301	766	90.7	10.48
	ORF2	+	271–741	471	156	16.8	6.15
	ORF3	+	2498–3058	561	186	20.8	11.21
TTV HNPP2	ORF1	+	596–2833	2238	745	88.7	10.28
	ORF2	+	237–728	492	163	17.2	7.18
TTV HNPP3	ORF1	+	596–2830	2235	744	88.5	10.29
	ORF2	+	237–728	492	163	17.0	7.38
TTV HNPP4	ORF1	+	595–2847	2253	750	89.1	10.41
	ORF2	+	260–721	462	153	16.7	6.79
TTV HNPP5	ORF1	+	592–2964	2373	790	92.4	10.69
	ORF2	+	260–733	474	157	16.9	7.22
TTV HNPP6-1	ORF1	+	587–2797	2211	736	85.9	10.50
	ORF2	+	237–710	474	157	16.8	7.82
	ORF3	+	2809–2988	180	59	7.1	9.66
TTV HNPP6-2	ORF1	+	589–2799	2211	736	85.9	10.50
	ORF2	+	239–712	474	157	16.8	7.82
	ORF3	+	2811–2990	180	59	7.1	9.66
TTV HNPP7-1	ORF1	+	596–2881	2286	761	90.5	10.26
	ORF2	+	237–728	492	163	17.3	7.79
TTV HNPP7-2	ORF1	+	597–2834	2238	745	88.8	10.28
	ORF2	+	238–729	492	163	17.3	7.79

## Data Availability

The datasets analyzed during the current study are available from the corresponding authors upon reasonable request. All the sequences in this manuscript can be obtained from the NCBI database GenBank with the accession numbers PP374828–PP374836. (https://www.ncbi.nlm.nih.gov, accessed on 17 May 2024).

## Supplementary Materials

The following supporting information can be downloaded at https://www.mdpi.com/article/10.3390/v16071059/s1. Figure S1: Amino acid comparison of ORF1 among 9 novel TTV variants. The black boxes indicate unique sites or motifs; Table S1: Torque teno virus genome size, GC content and gene and protein sequence identities.

## References

[1] Varsani A., Opriessnig T., Celer V., Maggi F., Okamoto H., Blomström A.-L., Cadar D., Harrach B., Biagini P., Kraberger S. (2021). Taxonomic update for mammalian anelloviruses (family Anelloviridae). Arch. Virol..

[2] Cosentino M.A.C., D’arc M., Moreira F.R.R., Cavalcante L.T.d.F., Mouta R., Coimbra A., Schiffler F.B., Miranda T.d.S., Medeiros G., Dias C.A. (2022). Discovery of two novel Torque Teno viruses in Callithrix penicillata provides insights on Anelloviridae diversification dynamics. Front. Microbiol..

[3] Hsiao K.L., Wang L.Y., Lin C.L., Liu H.F. (2016). New Phylogenetic Groups of Torque Teno Virus Identified in Eastern Taiwan Indigenes. PLoS ONE.

[4] Mi Z., Yuan X., Pei G., Wang W., An X., Zhang Z., Huang Y., Peng F., Li S., Bai C. (2014). High-throughput sequencing exclusively identified a novel Torque teno virus genotype in serum of a patient with fatal fever. Virol. Sin..

[5] Spandole-Dinu S., Cimponeriu D., Stoica I., Apircioaie O., Gogianu L., Berca L.M., Nica S., Toma M., Nica R. (2022). Phylogenetic analysis of torque teno virus in Romania: Possible evidence of distinct geographical distribution. Arch. Virol..

[6] Tyschik E.A., Shcherbakova S.M., Ibragimov R.R., Rebrikov D.V. (2017). Transplacental transmission of torque teno virus. Virol. J..

[7] Vecchia A.D., Kluge M., Silva J.V.d.S.d., Comerlato J., Rodrigues M.T., Fleck J.D., da Luz R.B., Teixeira T.F., Roehe P.M., Capalonga R. (2013). Presence of Torque teno virus (TTV) in tap water in public schools from Southern Brazil. Food Environ. Virol..

[8] Nishizawa T., Okamoto H., Konishi K., Yoshizawa H., Miyakawa Y., Mayumi M. (1997). A novel DNA virus (TTV) associated with elevated transaminase levels in posttransfusion hepatitis of unknown etiology. Biochem. Biophys. Res. Commun..

[9] Okamoto H. (2009). History of discoveries and pathogenicity of TT viruses. Curr. Top. Microbiol. Immunol..

[10] Hino S., Miyata H. (2007). Torque teno virus (TTV): Current status. Rev. Med. Virol..

[11] Reshetnyak V.I., Maev I.V., Burmistrov A.I., Chekmazov I.A., Karlovich T.I. (2020). Torque teno virus in liver diseases: On the way towards unity of view. World J. Gastroenterol..

[12] Desai M.M., Pal R.B., Banker D.D. (2005). Molecular epidemiology and clinical implications of TT virus (TTV) infection in Indian subjects. J. Clin. Gastroenterol..

[13] Spandole S., Cimponeriu D., Berca L.M., Mihăescu G. (2015). Human anelloviruses: An update of molecular, epidemiological and clinical aspects. Arch. Virol..

[14] Wang D., Wang X.-W., Peng X.-C., Xiang Y., Song S.-B., Wang Y.-Y., Chen L., Xin V.W., Lyu Y.-N., Ji J. (2018). CRISPR/Cas9 genome editing technology significantly accelerated herpes simplex virus research. Cancer Gene Ther..

[15] Spezia P.G., Baj A., Drago Ferrante F., Boutahar S., Azzi L., Genoni A., Dalla Gasperina D., Novazzi F., Dentali F., Focosi D. (2022). Detection of Torquetenovirus and Redondovirus DNA in Saliva Samples from SARS-CoV-2-Positive and -Negative Subjects. Viruses.

[16] Tang X., Cai L., Meng Y., Xu J., Lu C., Yang J. (2020). Indicator Regularized Non-Negative Matrix Factorization Method-Based Drug Repurposing for COVID-19. Front. Immunol..

[17] Zhang Y., Lian B., Yang S., Huang X., Zhou Y., Cao L. (2023). Metabotropic glutamate receptor 5-related autoimmune encephalitis with reversible splenial lesion syndrome following SARS-CoV-2 vaccination. Medicine.

[18] Prince C., Bounoutas G., Zhou B., Raja W., Gold I., Pozsgai R., Thakker P., Boisvert N., Reardon C., Thurmond S. (2024). A novel functional gene delivery platform based on a commensal human anellovirus demonstrates transduction in multiple tissue types. bioRxiv.

[19] Blanc P.D., Seaton A. (2016). Pneumoconiosis Redux. Coal Workers’ Pneumoconiosis and Silicosis Are Still a Problem. Am. J. Respir. Crit. Care Med..

[20] Leonard R., Zulfikar R., Stansbury R. (2020). Coal mining and lung disease in the 21st century. Curr. Opin. Pulm. Med..

[21] Huang L.Y., Oystein Jonassen T., Hungnes O., Grinde B. (2001). High prevalence of TT virus-related DNA (90%) and diverse viral genotypes in Norwegian blood donors. J. Med. Virol..

[22] Leary T.P., Erker J.C., Chalmers M.L., Desai S.M., Mushahwar I.K. (1999). Improved detection systems for TT virus reveal high prevalence in humans, non-human primates and farm animals. J. Gen. Virol..

[23] Okamoto H., Takahashi M., Nishizawa T., Ukita M., Fukuda M., Tsuda F., Miyakawa Y., Mayumi M. (1999). Marked genomic heterogeneity and frequent mixed infection of TT virus demonstrated by PCR with primers from coding and noncoding regions. Virology.

[24] Kumar S., Stecher G., Tamura K. (2016). MEGA7: Molecular Evolutionary Genetics Analysis Version 7.0 for Bigger Datasets. Mol. Biol. Evol..

[25] Zhou Z.J., Qiu Y., Pu Y., Huang X., Ge X.Y. (2020). BioAider: An efficient tool for viral genome analysis and its application in tracing SARS-CoV-2 transmission. Sustain. Cities Soc..

[26] Rosario K., Duffy S., Breitbart M. (2012). A field guide to eukaryotic circular single-stranded DNA viruses: Insights gained from metagenomics. Arch. Virol..

[27] Zheng H., Ye L., Fang X., Li B., Wang Y., Xiang X., Kong L., Wang W., Zeng Y., Ye L. (2007). Torque teno virus (SANBAN isolate) ORF2 protein suppresses NF-κB pathways via interaction with IkappaB kinases. J. Virol..

[28] Focosi D., Spezia P., Macera L., Salvadori S., Navarro D., Lanza M., Antonelli G., Pistello M., Maggi F. (2020). Assessment of prevalence and load of torquetenovirus viraemia in a large cohort of healthy blood donors. Clin. Microbiol. Infect..

[29] Hettmann A., Demcsák A., Bach Á., Decsi G., Dencs Á., Pálinkó D., Rovó L., Nagy K., Minarovits J., Takács M. (2016). Detection and Phylogenetic Analysis of Torque Teno Virus in Salivary and Tumor Biopsy Samples from Head and Neck Carcinoma Patients. Intervirology.

