# Supplementary material for: Detection and Genomic Characterization of Torque Teno Virus in Pneumoconiosis Patients in China
Source: Viruses. 2024 Jun 30;16(7):1059. doi: 10.3390/v16071059 (PMC11281462; doi:10.3390/v16071059)
Supplement: Supplementary file 1 [file viruses-16-01059-s001.zip › Table S1.pdf]

Table S1 Torque teno virus genome size, GC content, gene and protein sequence identities.

Torque teno virus	Genome size(nt)	G + C content (%)	pairwise identities of nucleotide and amino acid sequence(nt/aa%)																	
			TTV HNPP1		TTV HNPP2		TTV HNPP3		TTV HNPP4		TTV HNPP5		TTV HNPP6-1		TTV HNPP6-2		TTV HNPP7-1		TTV HNPP7-2	
			ORF1	ORF2	ORF1	ORF2	ORF1	ORF2	ORF1	ORF2	ORF1	ORF2	ORF1	ORF2	ORF1	ORF2	ORF1	ORF2	ORF1	ORF2
TTV HNPP1	3830	53.3	/	/	55/44	58/42	55/45	59/40	52/43	51/40	47/36	52/42	47/36	46/30	47/36	46/30	54/43	57/40	55/44	57/40
TTV HNPP2	3794	52.5	55/44	58/42	/	/	88/88	93/90	59/51	54/39	49/39	51/38	49/36	50/37	49/36	50/37	90/90	89/83	92/92	89/83
TTV HNPP3	3741	52.7	55/45	59/40	88/88	93/90	/	/	58/52	55/41	48/38	51/38	49/36	50/35	49/36	50/35	88/87	88/81	90/89	88/81
TTV HNPP4	3769	50.7	52/43	51/40	59/51	54/39	58/52	55/41	/	/	47/36	54/39	46/35	48/36	46/35	48/36	58/50	55/41	59/51	55/41
TTV HNPP5	3908	53.4	47/36	52/42	49/39	51/38	48/38	51/38	47/36	54/39	/	/	46/38	49/33	46/38	49/33	48/37	53/39	49/39	53/39
TTV HNPP6-1	3719	52.0	47/36	46/30	49/36	50/37	49/36	50/35	46/35	48/36	46/38	49/33	/	/	100/100	100/100	48/35	49/36	49/36	49/36
TTV HNPP6-2	3773	52.2	47/36	46/30	49/36	50/37	49/36	50/35	46/35	48/36	46/38	49/33	100/100	100/100	/	/	48/35	49/36	49/36	49/36
TTV HNPP7-1	3798	53.3	54/43	57/40	90/90	89/83	88/87	88/81	58/50	55/41	48/37	53/39	48/35	49/36	48/35	49/36	/	/	97/97	100/100
TTV HNPP7-2	3796	52.9	55/44	57/40	92/92	89/83	90/89	88/81	59/51	55/41	49/39	53/39	49/36	49/36	49/36	49/36	97/97	100/100	/	/
TTV CTBG012	3686	52.2	47/35	47/34	48/35	51/37	48/35	50/36	46/34	49/35	45/38	52/39	93/94	92/87	93/94	92/87	48/35	50/38	48/36	50/38
TCHN-G2	3214	51.2	54/44	58/40	88/92	88/83	87/89	88/80	56/51	55/41	47/38	53/39	47/36	49/35	47/36	49/35	92/90	98/97	90/93	98/97
SAfiA-235-4	3691	51.7	53/43	52/36	59/51	58/42	58/52	59/43	80/82	77/66	48/38	53/37	46/36	49/32	46/36	49/32	59/50	59/44	60/52	59/44
SAfiA-789-1	3636	51.7	47/35	46/32	47/36	51/39	48/37	51/37	45/35	48/35	45/38	50/36	91/96	94/89	91/96	94/89	48/35	50/39	47/36	50/39
SAfiA-320-7	3029	51.1	45/35	47/32	44/36	51/39	45/36	50/37	43/35	49/35	43/38	50/36	86/95	93/89	87/95	93/89	45/35	50/39	45/36	50/39
SAfiA-124-19	3016	51.9	44/35	42/40	46/38	42/33	46/38	41/33	44/36	44/37	84/94	77/91	44/38	38/28	44/38	38/28	47/37	43/35	46/37	43/35
SAfiA-461-2	3595	51.2	52/43	52/37	57/51	58/43	56/51	58/43	77/82	78/66	47/39	53/38	45/37	49/32	45/36	49/32	58/50	59/45	57/51	59/45
SAfiA-228-0	3578	51.2	52/44	57/40	85/93	89/83	83/91	88/82	54/52	55/40	46/38	52/40	46/36	49/35	46/36	49/35	88/92	97/97	86/95	97/97
hb036-anello-1	3721	52.9	89/88	97/94	54/42	59/42	54/44	59/42	52/42	51/40	46/35	52/42	47/34	45/31	47/34	45/31	54/43	58/42	54/43	58/42
Torque teno virus 19	3808	53.8	55/44	57/40	90/93	89/84	86/88	89/83	59/52	55/41	49/39	53/41	49/37	50/36	49/37	50/36	89/88	87/83	89/91	87/83
Torque teno virus 3	3103	50.7	44/35	48/34	43/35	53/40	43/35	52/37	42/35	48/33	41/37	51/39	64/66	76/66	64/66	76/66	44/35	52/40	43/36	52/40
Torque teno virus 16	3818	51.1	48/39	48/35	53/44	49/37	53/43	49/37	51/42	68/59	46/37	49/37	47/35	47/37	47/35	47/40	52/43	50/38	53/44	50/39
Torque teno virus 24	3246	52.6	85/85	80/96	52/43	48/37	53/44	48/39	51/42	41/38	46/35	42/41	45/33	38/31	45/33	38/31	54/43	47/38	53/43	47/38
