# Supplementary material for: Detection and Genomic Characterization of Torque Teno Virus in Pneumoconiosis Patients in China
Source: Viruses. 2024 Jun 30;16(7):1059. doi: 10.3390/v16071059 (PMC11281462; doi:10.3390/v16071059)

Figure S1. Amino acid comparison of ORF1 among 9 novel TTV. The black boxes indicate unique sites or motif.

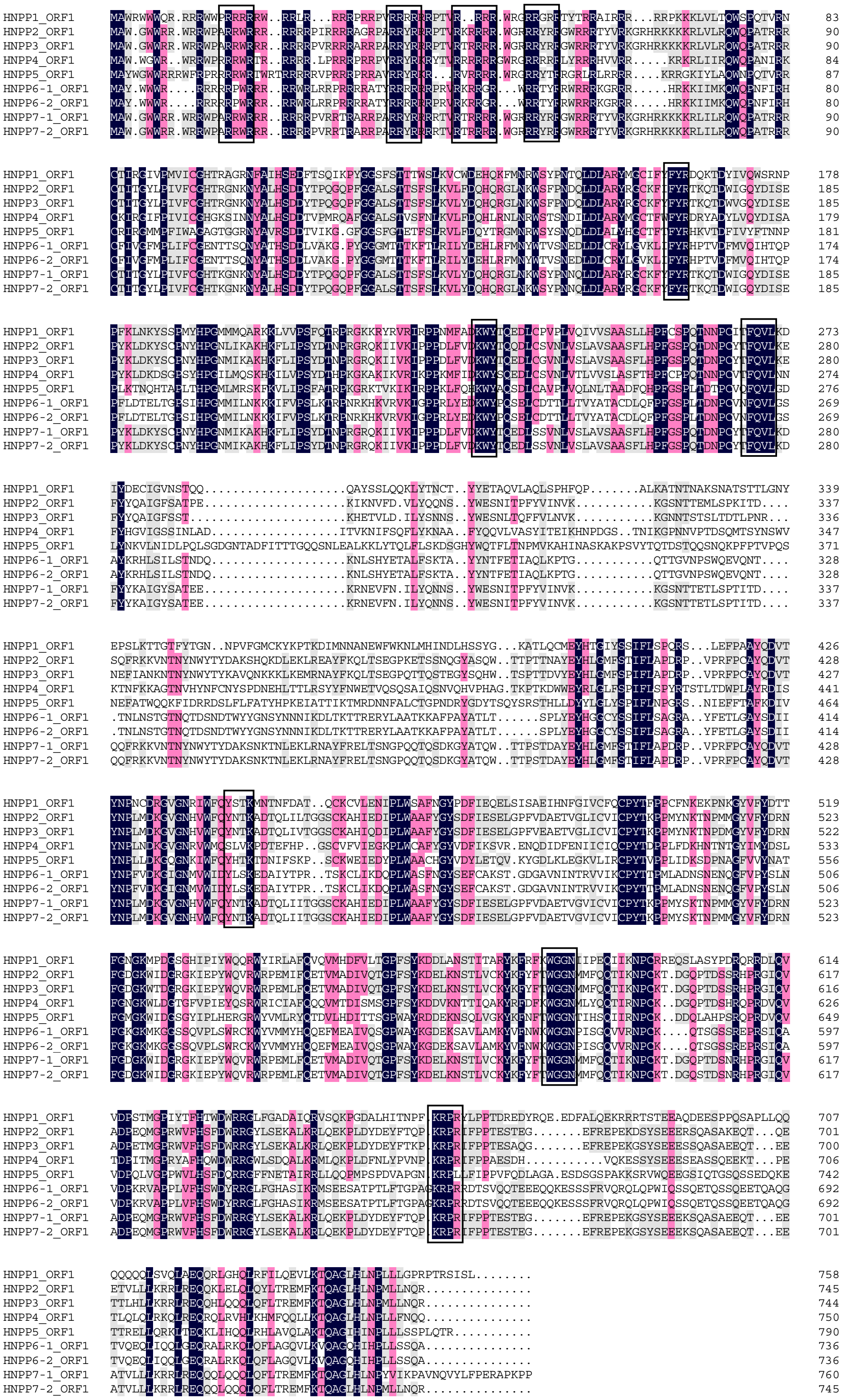

Supplement: Supplementary file 1 [file Figure S1.pdf › Figure S1.pdf]
